# The Dual Anaplerotic Model (DAM): Integral Roles of Pyruvate Carboxylase and the GABA Shunt in Beta Cell Insulin Secretion

**DOI:** 10.3390/life16010171

**Published:** 2026-01-20

**Authors:** Vladimir Grubelnik, Jan Zmazek, Marko Marhl

**Affiliations:** 1 Faculty of Electrical Engineering and Computer Science, University of Maribor, Koroška Cesta 46, 2000 Maribor, Slovenia; vlado.grubelnik@um.si; 2 National Institute of Public Health, Trubarjeva Cesta 2, 1000 Ljubljana, Slovenia; jan.zmazek@um.si; 3 Faculty of Natural Sciences and Mathematics, University of Maribor, Koroška Cesta 160, 2000 Maribor, Slovenia; 4 Faculty of Medicine, University of Maribor, Taborska Ulica 8, 2000 Maribor, Slovenia; 5 Faculty of Education, University of Maribor, Koroška Cesta 160, 2000 Maribor, Slovenia

**Keywords:** beta cell oscillations, mitochondrial metabolism, anaplerosis, cataplerosis, GABA shunt, PEP cycle

## Abstract

We present a simplified phenomenological computational framework that integrates the GABA shunt into established metabolic mechanisms underlying pancreatic beta cell insulin secretion. The GABA shunt introduces carbon into the tricarboxylic acid (TCA) cycle via succinate, thereby functioning as an anaplerotic pathway. This anaplerotic input is coupled to oscillatory cataplerotic fluxes, primarily involving α-ketoglutarate, whose effective extrusion requires coordinated counter-fluxes of malate and aspartate. Within the model, these cataplerotic exchanges are facilitated by UCP2-mediated transport processes and necessitate complementary anaplerotic replenishment through pyruvate carboxylase (PC). Based on this functional interdependence, we introduce the Dual Anaplerotic Model (DAM), which conceptually links two anaplerotic routes—the GABA shunt-mediated pathway and the glucose-dependent PC pathway—into a unified metabolic response module. DAM describes a coordinated, breathing-like redistribution of carbon between mitochondrial and cytosolic metabolite pools, while efficient oxidative metabolism of glucose-derived carbon entering the TCA cycle via pyruvate dehydrogenase is maintained. The model is driven by experimentally observed ATP/ADP and Ca^2+^ dynamics and is not intended to generate autonomous oscillations. Instead, it enables qualitative, phase-dependent visualization of how dual anaplerotic fluxes constrain and shape oscillatory metabolic states in beta cells. DAM provides an integrative conceptual scaffold for interpreting experimental observations and for motivating future quantitative modeling and experimental studies addressing metabolic regulation in physiological and pathophysiological contexts.

## 1. Introduction

Insulin secretion from pancreatic islet beta cells occurs in a pulsatile manner, with a characteristic periodicity of approximately five minutes [1]. The mechanistic basis of this oscillatory behavior has been investigated for several decades, resulting in a wide range of conceptual and mathematical models. These models differ substantially in their proposed sources of rhythmogenesis and regulatory mechanisms, yet most share a central role of ATP production and the ATP-dependent closure of ATP-sensitive potassium (K_ATP_) channels—a concept has been experimentally established and progressively refined.

One of the most comprehensive frameworks is the Dual Oscillator Model (DOM), which was developed to explain the coupling between metabolic and electrical activity in beta cells. DOM integrates a glycolytic oscillator, driven by feedback regulation of phosphofructokinase (PFK) by fructose-1,6-bisphosphate (FBP), with an electrical oscillator, based on calcium-dependent ion channel dynamics [2]. This framework successfully reproduces a wide spectrum of experimentally observed behaviors, including fast, slow, and compound oscillations. A major advancement came from experimental findings showing that glycolytic intermediates, particularly FBP, oscillate in antiphase with the well-documented Ca^2+^ oscillations [3]. This discovery led to the development of a modified DOM [4], which attributes slow islet oscillations to the interplay between glycolytic dynamics and Ca^2+^ feedback. In this model, Ca^2+^ regulates pyruvate dehydrogenase (PDH) and stimulates ATP hydrolysis; together, these interactions modulate ATP production and consequently influence the cell’s electrical activity.

Building on this foundation, the Integrated Oscillator Model (IOM) was introduced by Bertram et al. (2018) [5], incorporating feedback regulation of glycolytic flux by cytosolic Ca^2+^ via PDH activity. In this framework, low Ca^2+^ levels reduce glycolytic efflux, leading to FBP accumulation, whereas high Ca^2+^ levels enhance efflux, resulting in FBP depletion. These dynamics closely match experimentally observed FBP oscillations [3], reinforcing the view that glycolysis and calcium signaling are tightly coupled in beta cell oscillations.

More recently, additional models have emphasized the importance of mitochondrial metabolism—particularly the coordinated roles of anaplerosis, cataplerosis, and oxidative phosphorylation (OxPhos)—in shaping oscillatory insulin secretion. The Mito_Cat_–Mito_Ox_ framework [6] extends earlier approaches by incorporating both temporal and spatial aspects of beta cell metabolism. This model integrates the roles of anaplerosis, pyruvate kinase (PK) activity, and the mitochondrial phosphoenolpyruvate (PEP) cycle into a comprehensive framework of glucose-stimulated insulin secretion (GSIS). It proposes that during the Mito_Cat_ phase, PK elevates ATP/ADP ratios in the plasma membrane microdomain, leading to closure of K_ATP_ channels and membrane depolarization. In contrast, during the Mito_Ox_ phase, mitochondrial OxPhos is activated in response to rising ADP levels, generating ATP to meet the energetic demands of insulin exocytosis and ion transport. Viewed more broadly, the Mito_Cat_–Mito_Ox_ model acknowledges that multiple interconnected metabolic and ionic processes exhibit phase-specific acceleration and deceleration—coinciding with either the electrically silent triggering phase (Mito_Cat_) or the electrically active secretory phase (Mito_Ox_) of insulin oscillations.

Our group has also contributed to this field by developing a computational model that integrates anaplerotic metabolism, localized ATP production, and redox signaling to simulate beta cell responses to both glucose and combined glucose-glutamine stimulation [7]. This model extends beyond a sole focus on OxPhos and K_ATP_ channel activity by emphasizing localized ATP generation from PEP in proximity to K_ATP_ channels. It also highlights the signaling role of hydrogen peroxide (H_2_O_2_) during the first phase of insulin secretion and underscored the importance of anaplerotic metabolism during the second phase—particularly the production of NADPH and glutamate (Glu) as key amplifiers of insulin release. In a subsequent study [8], we further distinguished between spatially and functionally distinct ATP pools and separately analyzing the first triggering phase and the second amplifying phase of beta cell activation.

In addition to these efforts, several other models of beta cell dynamics have been proposed; for a recent comprehensive review, see [9]. Notably, recent advances increasingly emphasize the importance of the GABA shunt in beta cell metabolism. The GABA shunt has long been recognized as a pathway through which GABA-derived succinate (Succ) enters the tricarboxylic acid (TCA) cycle, thereby providing additional carbon and increasing TCA flux [10,11]. More recently, it has been identified as a significant anaplerotic contributor to beta cell metabolism and insulin secretion [12]. Despite this recognition, to our knowledge no mathematical or computational models have explicitly integrated the GABA shunt into frameworks describing beta cell metabolic dynamics.

Here, we therefore aim to construct an integrative conceptual scaffold that captures how the GABA shunt contributes anaplerotic carbon to the TCA cycle. While this anaplerotic input occurs via Succ, it must be balanced by cataplerotic fluxes, primarily involving α-ketoglutarate (αKG), whose effective extrusion requires coordinated counter-fluxes of C4 metabolites, particularly malate (Mal) and aspartate (Asp). Consequently, the operation of the GABA shunt cannot be considered in isolation. In the present framework, these cataplerotic exchanges require complementary anaplerotic replenishment via pyruvate carboxylase (PC), most likely supported by uncoupling protein 2 (UCP2).

Based on this functional interdependence, we introduce the Dual Anaplerotic Model (DAM), which conceptually links two anaplerotic routes—the GABA shunt-mediated pathway and the glucose-dependent PC pathway—into a unified metabolic response module. DAM describes a coordinated, breathing-like redistribution of carbon between mitochondrial and cytosolic metabolite pools, while maintaining efficient oxidative metabolism of glucose-derived carbon entering the TCA cycle via PDH.

The DAM presented here is a simplified phenomenological scaffold driven by experimentally observed ATP/ADP and Ca^2+^ dynamics and is not intended to generate autonomous oscillations. Instead, it enables qualitative, phase-dependent visualization of how dual anaplerotic fluxes constrain and shape oscillatory metabolic states in beta cells. Within a Mito_Cat_–Mito_Ox_ framework, DAM offers a simplified synergistic view in which PC- and GABA shunt-driven anaplerotic routes function as interdependent and temporally coordinated processes: the GABA shunt predominantly reinforcing the Mito_Ox_ phase, and PC-derived flux contributing not only to PEP cycling but also to cataplerotic replenishment of the GABA pool during the Mito_Cat_ phase. In this way, DAM provides an integrative conceptual scaffold for interpreting experimental observations and for motivating future quantitative modeling and experimental studies addressing metabolic regulation under physiological and pathophysiological conditions.

In the following sections, we first introduce the model conceptually and describe its qualitative operation. The system is represented by four core metabolic pools, and the key processes governing fluxes between them are outlined. The mathematical formulation is deliberately kept minimal to highlight temporal transitions and phase-specific activation patterns—particularly the cyclic emptying and refilling of metabolite pools. To complement the mathematical description, we also provide an animation illustrating the oscillatory behavior of the principal pools. Finally, we discuss how the model’s predictions relate to available experimental data and how this minimal framework may serve as a foundation for future extensions toward more comprehensive and physiologically detailed models of glucose-stimulated beta cell function.

## 2. Model

In this model, beta cell metabolism is conceptualized as a synergistic interplay among glycolysis, the TCA cycle, the PEP cycle, and the GABA shunt. To reduce biochemical complexity while retaining essential functional relationships, the system is represented using a four-pool framework. Each pool in this framework represents a coarse-grained metabolite pool, in which multiple biochemically related intermediates are grouped into a single effective state variable. This coarse-graining is introduced to reduce biochemical detail while preserving the dominant flux pathways, phase relationships, and functional coupling between glycolysis, mitochondrial metabolism, and the GABA shunt. Within each pool, individual metabolites are assumed to remain in relative equilibrium, such that the pool dynamics reflect net carbon redistribution rather than the kinetics of specific enzymatic steps.

The first pool, P_0_, comprises downstream glycolytic intermediates, primarily FBP and PEP. These metabolites are direct precursors of pyruvate (Pyr), which serves as the principal source of carbon entering mitochondrial metabolism.

The second metabolite pool, P_1_, corresponds to a coarse-grained representation of the right half of the TCA cycle and includes citrate (Cit), isocitrate (Isocit), and αKG. This pool effectively represents the primary site of carbon entry into the TCA cycle via the condensation of acetyl-CoA with oxaloacetate (OAA).

The third metabolite pool, P_2_, represents a coarse-grained left half of the TCA cycle, comprising primarily C_4_ dicarboxylates such as malate and fumarate (Fum). Mal, in particular, plays a dominant role in redox balance and metabolite transport, while other intermediates are assumed to be in near-equilibrium with it.

The fourth pool, P_3_, captures the GABA reservoir, which is tightly linked to TCA cycle metabolism through the GABA shunt. This pathway provides an alternative anaplerotic input into the TCA cycle via Succ production, especially under conditions that favor increased GABA flux, such as elevated glucose availability or glutamine (Gln) co-stimulation.

**Figure 1:**
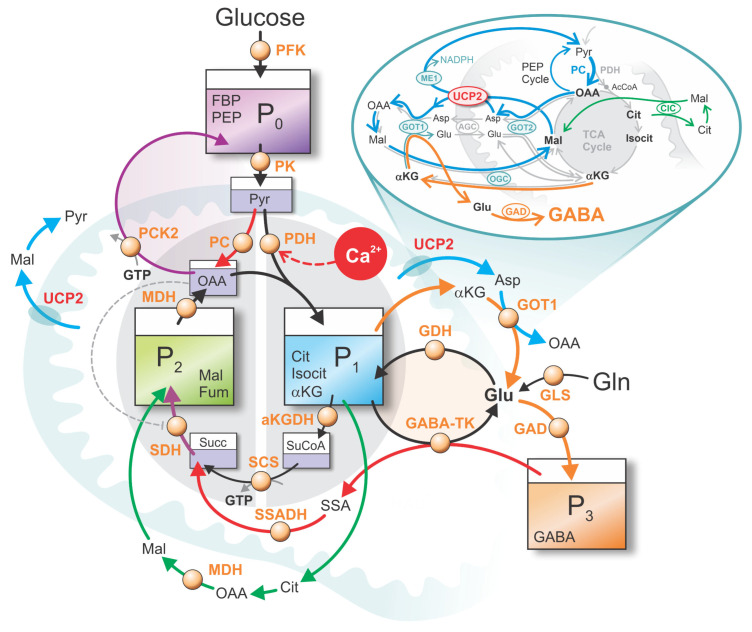
Schematic representation of the glucose-stimulated beta cell as a four-pool model: $P_{0}$—Downstream glycolytic pool, $P_{1}$—Right half of the TCA cycle, $P_{2}$—Left half of the TCA cycle, $P_{3}$—GABA pool. Solid arrows indicate metabolic fluxes, whereas dashed lines denote regulatory influences acting on specific fluxes. Arrow colors are chosen for visual clarity and to aid pathway tracking; individual colors are used consistently to associate related metabolic pathways and cyclic processes but do not carry quantitative meaning. Abbreviations used in the figure: AcCoA—acetyl-coenzyme A, AGC—aspartate–glutamate carrier, αKG—α-ketoglutarate, αKGDH—α-ketoglutarate dehydrogenase complex, ATP—adenosine-triphosphate, Asp—aspartate, CIC—citrate carrier, Cit—citrate, FBP—fructose-1,6-bisphosphate, Fum—fumarate, GABA—γ-aminobutyric acid, GABA-TK—GABA transaminase, GAD—glutamate decarboxylase, GDH—glutamate dehydrogenase, Glu—glutamate, Gln—glutamine, GOT1—glutamic-oxaloacetic transaminase 1, GOT2—glutamate–oxaloacetate transaminase 2, GLS—glutaminase, GTP—guanosine-triphosphate, Isocit—isocitrate, Mal—malate, ME1—malic enzyme, MDH—malate dehydrogenase, NADPH—nicotinamide adenine dinucleotide phosphate, OAA—oxaloacetate, OGC—oxoglutarate carrier, PC—pyruvate carboxylase, PCK2—phosphoenolpyruvate carboxykinase (mitochondrial isoform), PDH—pyruvate dehydrogenase, PEP—phosphoenolpyruvate, PFK—phosphofructokinase, PK—pyruvate kinase, Pyr—pyruvate, SCS—succinyl-CoA synthetase, SDH—succinate dehydrogenase, SSA—succinic semialdehyde, SSADH—succinic semialdehyde dehydrogenase, Succ—succinate, SuCoA—succinyl-CoA, UCP2—uncoupling protein 2.

The main fluxes between pools P_0_–P_3_ are schematically illustrated in Figure 1. A central flux connects P_0_ to P_1_ via PDH, representing the Ca^2+^-sensitive entry of Pyr into the TCA cycle through acetyl-CoA. Elevated Ca^2+^ enhances PDH activity, thereby facilitating carbon flow from P_0_ to P_1_ and simultaneously from P_2_ to P_1_, as one OAA combines with one acetyl-CoA to form Cit in P_1_. This reaction enables oxidative metabolism, defining the Mito_Ox_ phase [13,14].

The GABA pool (P_3_) contributes to the TCA cycle through the GABA shunt, an anaplerotic pathway (highlighted in red in Figure 1) that generates Succ and feeds it into P_2_. This flux from P_3_ to P_2_ provides a source of “fresh carbon” to the cycle and plays a crucial role in enhancing the Mito_Ox_ phase [12].

A second, well-established anaplerotic flux—also highlighted in red in Figure 1—is the glucose-derived entry via PC, which channels Pyr into the PEP cycle and contributes to OAA replenishment. This PC-derived OAA is required not only for sustaining TCA cycle intermediates but also for Asp formation and extrusion, a process that directly interfaces with GABA metabolism. Together, PC- and GABA shunt-driven pathways form tightly interdependent and temporally coordinated anaplerotic routes (see inset of Figure 1).

Because both GABA-mediated and PC-mediated fluxes are anaplerotic, they must be balanced by cataplerotic fluxes to maintain near-zero net carbon flux and allow metabolite concentrations to oscillate around quasi-stationary values. Cataplerotic fluxes, however, are inherently constrained: many metabolites—including Mal, Cit, and αKG—predominantly undergo compartmental exchange rather than true net efflux [6]. Genuine net carbon export from the mitochondrial matrix occurs only through regulated transport processes. In the present model, this cataplerotic exchange is represented by a redox-dependent export term primarily motivated by UCP2-associated C_4_ metabolite transport, as supported by experimental evidence reported by Vozza et al. (2014) [15]. In particular, this process was shown to be tightly regulated by GTP levels and co-regulated with the reduced quinone pool (Q_red_) [16]. At the same time, we explicitly acknowledge that additional transport pathways—such as phosphate-linked exchangers (DIC, PIC)—may contribute to C_4_ exchange, as analyzed in detail in the Supplementary Material of our previous work [7].

Within the present framework, the principal net cataplerotic flux corresponds to carbon redistribution from pool $P_{1}$ into pool $P_{3}$ via cytosolic Glu production and its subsequent conversion to GABA, as illustrated in the inset of Figure 1. A critical mechanistic element of this pathway is the export of Asp into the cytosol, which provides the necessary amino-group transfer to αKG via transamination, thereby enabling cytosolic Glu synthesis. Glu subsequently serves both as a precursor for GABA production and as a means of replenishing the GABA pool ($P_{3}$). In this sense, UCP2-facilitated C_4_ extrusion represents a necessary condition for achieving net cataplerotic flow from $P_{1}$ to $P_{3}$ within the present modeling framework, thereby completing the cataplerotic arm of the GABA shunt.

Importantly, this replenishment of the GABA pool is critically supported by simultaneous PC-derived anaplerotic flux. PC activity sustains OAA availability for Asp formation and extrusion and, via Mal export and its conversion back to Pyr by cytosolic malic enzyme (ME1), closes the anaplerotic–cataplerotic cycle. Together, the PC- and GABA shunt-driven pathways operate as interdependent and temporally coordinated processes that enable efficient redistribution of carbon across pools while preserving oxidative metabolism. In parallel, redistribution of Cit from $P_{1}$ into Mal in $P_{2}$ via the Cit–Mal exchanger (CIC) is also represented in Figure 1, further illustrating the interconnected nature of these fluxes.

The model dynamics is based on experimentally measured Ca^2+^ and ATP traces. Several studies have shown that, in pancreatic beta cells, ATP and Ca^2+^ oscillations occur in opposite phase [17,18,19,20]. Specifically, ATP levels are maximal when Ca^2+^ concentration is minimal, whereas ATP reaches its minimum shortly before the Ca^2+^ peak [18].

**Figure 2:**
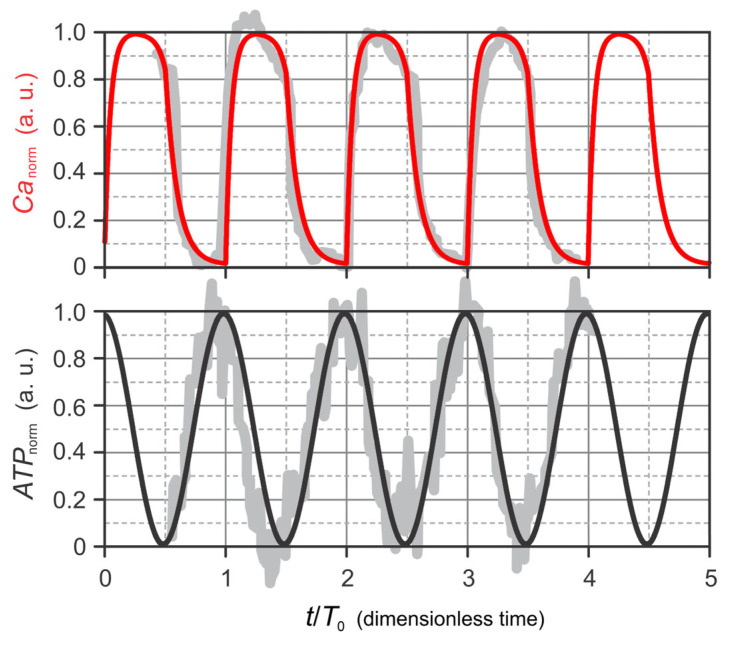
Fitting of mathematical functions to experimental data. Gray lines represent experimental data from Gregg et al. (2019) [19]; the red curve shows the fitted mathematical function for Ca^2+^ dynamics (Equation (1)), and the black curve shows the fitted function for ATP concentration (Equation (2)).

Here, we adopt experimental measurements from Gregg et al. (2019) [19] as the basis for modeling Ca^2+^ and ATP dynamics. Because the present model is a phenomenological framework aimed at visualizing phase-dependent relationships among model variables rather than reproducing absolute concentrations, the experimentally obtained Ca^2+^ and ATP traces were normalized to the interval $\left[0,1\right]$ (Figure 2). The resulting normalized variables, denoted $Ca_{norm}$ and ${ATP}_{norm}$, serve as external regulatory inputs to the model. The fitted functional forms are given by the following equations:

(1) $$\frac{d{Ca}_{norm}}{dt}=\left\{\begin{array}{cc} k_{0}\left(1-Ca_{norm}\right), & mod\left(t,T_{0}\right)<t_{1}\\ -k_{1}\left(1-Ca_{norm}\right), & t_{1}<mod\left(t,T_{0}\right)<t_{2}\\ -k_{2}Ca_{norm}, & mod\left(t,T_{0}\right)>t_{2} \end{array}\right.,$$

(2) $${ATP}_{norm}=A_{0}+A_{1}\left(1+cos\;\left(2\pi \frac{t}{T_{0}}\right)\right),$$

where both $Ca_{norm}$ and $ATP_{norm}$ are dimensionless variables confined to the interval $\left[0,1\right]$. Consequently, time t and all parameters are expressed in dimensionless units. The parameter values used are $k_{0}=22$, $k_{1}=15$, $k_{2}=10$, $t_{1}=0.25$, $t_{2}=0.5$, $T_{0}=1$, $A_{0}=0$, and $A_{1}=0.5$.

As presented in Figure 1, we consider the system as a four-pool model, for simplicity grouping together the key metabolites into the glycolytic compartment, the “left” part of the TCA cycle, the “right” part of the TCA cycle, and the GABA pool. Mathematically, we assume that the concentrations of the metabolites within each pool oscillate simultaneously; although their absolute values differ, their temporal dynamics are considered to be in phase. In the mathematical formulation, we define the four pools as follows:

(3) $$P_{0}=\left\{FBP,\;PEP\right\},\;P_{1}=\left\{Cit,\;Isocit,\;aKG\right\},\;P_{2}=\left\{Mal,\;\;Fum\right\},\;P_{3}=\left\{GABA\right\}.$$

To mathematically describe the dynamic behavior of metabolite pools involved in GSIS, we developed a minimal model based on four interacting metabolic pools. These pools represent key components of beta cell metabolism: glycolysis ($P_{0}$), the right-hand segment of the TCA cycle ($P_{1}$), the left-hand segment of the TCA cycle ($P_{2}$), and the GABA shunt ($P_{3}$). Each pool comprises a representative group of metabolites that are assumed to oscillate with broadly similar temporal dynamics, while the fluxes between them are governed by biologically motivated, nonlinear expressions.

Rather than aiming for molecular-level biochemical detail, the model prioritizes conceptual simplicity while retaining physiological relevance. Fluxes are formulated using multiplicative power-law expressions, in which each flux depends on the concentrations of key regulatory variables—typically as products of variables raised to fixed exponents. This structure captures essential features of metabolic control, including Ca^2+^-dependent activation, redox-mediated feedback, and ATP-dependent regulation, while keeping the model analytically transparent and computationally tractable.

Both ATP and NADH are central indicators of the cellular energetic and redox state and act as activators or inhibitors in multiple metabolic pathways. In the present phenomenological framework, we do not distinguish between their detailed kinetics. Instead, we assume that ATP/ADP and NADH/NAD^+^ ratios rapidly equilibrate via mitochondrial OxPhos, such that their temporal dynamics are strongly correlated. Accordingly, we introduce a dimensionless, normalized redox–energy proxy $NADH_{norm}(t)\in [0,1]$, which represents the normalized cellular reducing state rather than the concentration of NADH itself.

Because the experimentally measured ATP trace is used as the primary energetic input to the model, the same normalized signal is reused as a proxy for redox-dependent regulation. Thus, within the scope of this minimal model, we define:

(4) $${NADH}_{norm}(t)\equiv {ATP}_{norm}(t).$$

where both variables are dimensionless and confined to the interval $\left[0,1\right]$. This identification reflects correlated energetic and redox states rather than molecular identity and allows redox-sensitive regulatory effects to be incorporated without introducing additional dynamical variables.

The resulting system of model equations offers a mechanistic yet minimal framework for simulating the oscillatory metabolic dynamics that underpin insulin secretion. In the sections below, we define the dynamics of each pool and the fluxes that interconnect them.

**Figure 3:**
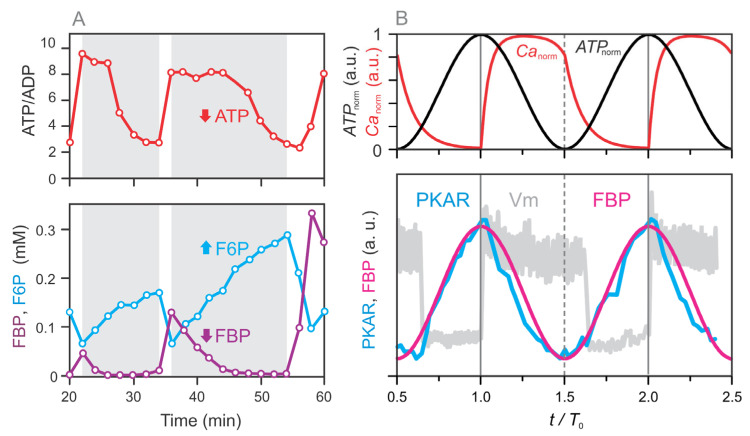
FBP dynamics is synchronized with ATP oscillations. (**A**) Phase annotation of experimental data from Tornheim (1997) [21], highlighting the synchronous oscillatory behavior of ATP (measured as the ATP/ADP ratio) and FBP. White-shaded regions indicate phases during which both ATP and FBP concentrations increase, while gray-shaded regions denote phases of concurrent decline. In contrast, F6P dynamics is in anti-phase with FBP. (**B**) Fit of the model-based FBP dynamics (Equation (5)) to experimental data extracted from Merrins et al. (2016) [3], demonstrating that FBP oscillations exhibit a smooth, sinusoidal-like profile closely aligned with ATP dynamics. The gray curve labeled Vm represents the experimentally measured membrane potential, included for orientation only, to indicate the timing of electrical activity relative to metabolic oscillations.

The dynamics of the glycolytic pool ($P_{0}$) is modeled based on experimental data for FBP reported by Tornheim (1997) [21]. As shown in Figure 3A, we extracted these data and annotated regions (white and gray shaded) to indicate phases during which FBP and ATP exhibit synchronous behavior. Specifically, both FBP and ATP increase during the white regions and decrease during the gray regions, demonstrating that their oscillations are in phase.

Although the waveform profiles of FBP and ATP are not identical, more recent studies—specifically in pancreatic beta cells [3]—have shown that FBP oscillations adopt a smoother, more sinusoidal-like profile that closely resembles ATP dynamics. Based on these findings, we approximate the FBP dynamics using a sinusoidal function fitted to the experimental data. Figure 3B compares the extracted data from Merrins et al. (2016) [3] with the sinusoidal function used in our model, showing good agreement.

Accordingly, the FBP-related glycolytic pool ($P_{0}$) is modeled as:

(5) $$P_{0}=k_{0}+k_{0,A}{ATP}_{norm},$$

where $k_{0}=0.1$, $k_{0,A}=0.2$.

The glycolytic pool ($P_{0}$) is tightly coupled to the TCA cycle through the generation of Pyr from the intermediates FBP and PEP. One major route for Pyr to enter the TCA cycle is via the oxidative pathway through PDH, which converts Pyr into acetyl-CoA. Acetyl-CoA subsequently combines with OAA, derived from Mal ($P_{2}$), to form Cit in the first TCA pool ($P_{1}$). The flux through PDH, denoted $J_{01}$, is regulated by several factors: the concentration of glycolytic intermediates ($P_{0}$) that give rise to Pyr [4,22], the availability of OAA as reflected by Mal levels in $P_{2}$ [22], and intracellular Ca^2+^, which stimulates PDH activity [13,14]. Although PDH is also inhibited by NADH—in other words, activated by the term ($1-{NADH}_{norm}$) in our model—Ca^2+^ and ($1-{NADH}_{norm}$) (which is related to $1-{ATP}_{norm}$) oscillate in phase (see Figure 2). This means that both signals would modulate $J_{01}$ in the same direction. To reduce redundancy while preserving physiologically relevant regulation, we simplify the model by including only Ca^2+^ as the modulating factor. This results in a reduced yet biologically meaningful representation of PDH regulation. The resulting flux $J_{01}$ is defined as:

(6) $$J_{01}=k_{01}{Ca}_{norm}P_{2}P_{0},$$

with $k_{01}=7$.

Given that Cit synthase consumes one molecule of acetyl-CoA and one molecule of OAA to generate Cit, and assuming a steady-state concentration of acetyl-CoA, the flux through Cit synthase ($J_{21}$) must equal the flux of acetyl-CoA production via PDH ($J_{01}$). This reflects the 1:1 stoichiometry of the reaction and allows us to equate these two fluxes:

(7) $$J_{21}=J_{01}.$$

Accordingly, the dynamics of pool $P_{1}$—representing intermediates in the “right-hand” side of the TCA cycle—is governed by:

(8) $$\frac{dP_{1}}{dt}=J_{21}-J_{12}-J_{13}.$$

The flux $J_{12}$ represents the carbon transfer from the “right” to the “left” side of the TCA cycle. This transfer encompasses both the canonical clockwise pathway through succinyl-CoA and Succ, as well as the cataplerotic–anaplerotic route mediated by Cit–Mal exchange. The latter pathway becomes particularly active under high-energy, nutrient-rich conditions, when excess Cit is exported from mitochondria to the cytosol via CIC (see inset of Figure 1).

Because the canonical oxidative pathway results in complete carbon loss as CO_2_ and does not contribute to net carbon redistribution between pools, it is not explicitly represented in the model. Accordingly, the flux $J_{12}$ is defined solely through the Cit–Mal exchange and is expressed as:

(9) $$J_{12}=k_{12}\cdot P_{1}\cdot {\left({NADH}_{norm}\right)}^{2}$$

Here, $P_{1}$ represents the dependence of the flux on metabolite availability in the right-hand segment of the TCA cycle, and the parameter value is set to $k_{12}=1$. The quadratic dependence on ${NADH}_{norm}$ ephasizes that $J_{12}$ is strongly regulated by the mitochondrial redox state. This nonlinearity is introduced phenomenologically to capture an effective switch-like regulation of Cit export under conditions of elevated reducing equivalents, particularly given that ${NADH}_{norm}$ is confined to the interval $\left[0,1\right]$.

Elevated NADH levels inhibit the clockwise TCA flux by suppressing the activity of Isocit dehydrogenase (IDH) and αKG dehydrogenase (αKGDH) [23,24,25,26]. This inhibition promotes Cit accumulation and its subsequent export from mitochondria to the cytosol, where Cit is cleaved into acetyl-CoA and OAA. The latter is then reduced by MDH, using NADH, to form Mal, which is subsequently transported back into the mitochondrial matrix (see the inset of Figure 1). Together, these processes justify the strong dependence of flux $J_{12}$ on the normalized NADH signal (${NADH}_{norm}$) used in the model.

Flux $J_{13}$ represents the production of GABA from αKG via the Mal–Asp shuttle and the C4 efflux mediated by UCP2 channels, as illustrated in greater detail in the inset of Figure 1. In the model, this flux is simplified by assuming dependence on the availability of αKG (represented by pool $P_{1}$) and primarily on the normalized redox state:

(10) $$J_{13}=k_{13}\cdot P_{1}\cdot {\left({NADH}_{norm}\right)}^{2}.$$

Because ${NADH}_{norm}$ is confined to the interval $\left[0,1\right]$, the quadratic dependence acts as a simple nonlinear weighting that emphasizes activation of this flux predominantly under highly reduced conditions, without introducing additional saturation parameters. The parameter value is set to $k_{13}=2$.

The dependence of $J_{13}$ on $NADH_{norm}$ reflects the assumption that mitochondrial cataplerotic exchange is coupled to the redox state and is represented in the model by a UCP2-associated C_4_ metabolite export term across the inner mitochondrial membrane. Elevated NADH levels are associated with a highly reduced mitochondrial state and increased reactive oxygen species (ROS) production, conditions known to activate UCP2 and promote C4 transport [15,27,28]. Importantly, αKG cannot be exported directly from the mitochondrial matrix; its effective utilization for cytosolic Glu and downstream GABA synthesis therefore requires obligatory counter-flux of C4 metabolites, primarily Mal and Asp.

In this framework, UCP2-mediated export of Asp into the cytosol supports amino-group transfer to αKG via transamination, thereby promoting cytosolic Glu production (see inset of Figure 1). Glu subsequently serves both as a precursor for GABA synthesis and as a means of replenishing the GABA pool ($P_{3}$). In this way, redox-dependent C4 extrusion constitutes a necessary condition for the net cataplerotic flow from $P_{1}$ to $P_{3}$, completing the cataplerotic arm of the GABA shunt.

The dynamics of pool $P_{2}$, which represents the metabolites in the left-hand segment of the TCA cycle, is described by the following equation:

(11) $$\frac{dP_{2}}{dt}=J_{12}-J_{21}+J_{32},$$

where the fluxes $J_{21}$ (Equation (7)) and $J_{12}$ (Equation (9)) have been defined previously.

The flux $J_{32}$ accounts for the anaplerotic input into the left-hand segment of the TCA cycle via the GABA shunt, with Succ as the key product replenishing the cycle. The GABA shunt is a three-step enzymatic pathway in which Glu is first converted to GABA by Glu decarboxylase (GAD), then to succinic semialdehyde by GABA transaminase (GABA-TK), and finally to Succ by succinic semialdehyde dehydrogenase (SSADH). This pathway bypasses the αKGDH step and provides an alternative route of carbon entry into the TCA cycle (see Figure 1).

Among the enzymes involved, SSADH is directly dependent on NAD^+^ and produces NADH in its final reaction step. While neither GAD nor GABA-TK consumes NADH directly, GAD activity has been reported to be sensitive to the cellular redox state, with elevated NADH levels inhibiting GABA formation [12]. In this way, the redox state of the cell indirectly regulates flux through the GABA shunt and, consequently, the magnitude of $J_{32}$.

Given that pancreatic beta cells express the full set of enzymes required for GABA shunt activity [29], this pathway is metabolically relevant under physiological conditions. In the present model, the flux $J_{32}$ is described as a function of the GABA pool ($P_{3}$), while incorporating redox-dependent inhibition via the normalized NADH signal:

(12) $$J_{32}=k_{32}\cdot P_{3}\cdot \left(1-{NADH}_{norm}\right).$$

with parameter value $k_{32}=1$.

Finally, the dynamics of the GABA pool ($P_{3}$) is modeled as:

(13) $$\frac{dP_{3}}{dt}=J_{13}-J_{32},$$

where flux $J_{13}$ is defined in Equation (10) and $J_{32}$ in Equation (12).

## 3. Results

**Figure 4:**
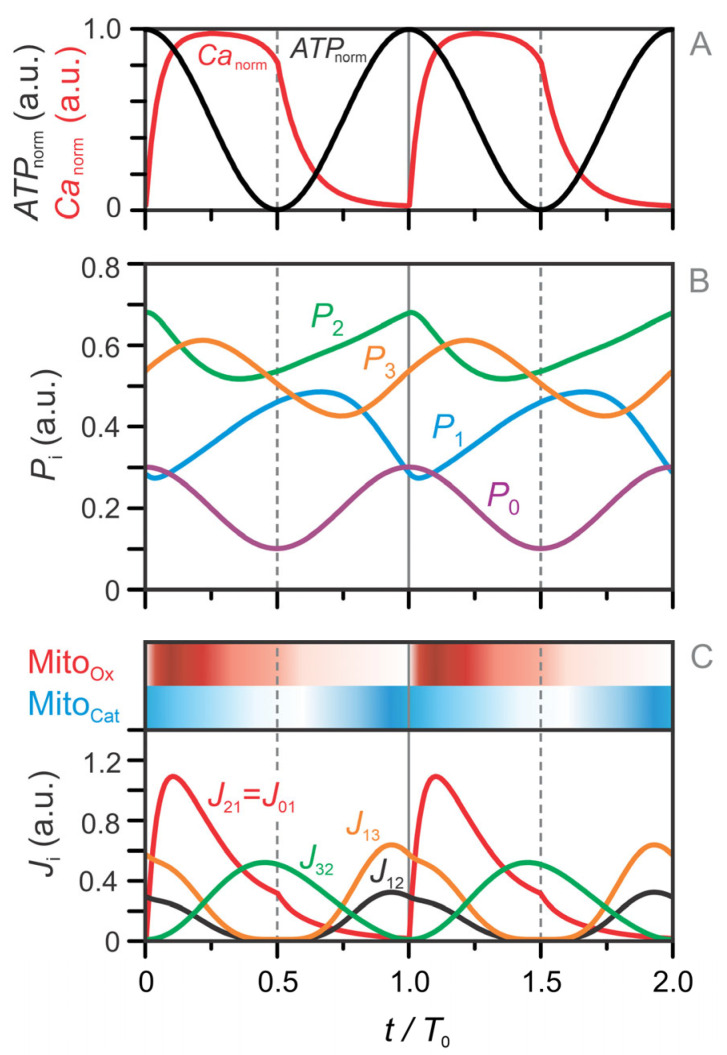
Simulated dynamics of metabolic pools ($P_{0}$–$P_{3}$) and the corresponding fluxes between them. (**A**) Time courses of ${Ca}_{norm}$ (red) and ${ATP}_{norm}$ (black), which serve as inputs to the model and are fitted to experimental data (see Figure 2 and Equations (1) and (2)). (**B**) Temporal dynamics of the four metabolic pools: the glycolytic pool ($P_{0}$), right-hand TCA cycle intermediates ($P_{1}$), left-hand TCA cycle intermediates ($P_{2}$), and the GABA pool ($P_{3}$). (**C**) Key fluxes between pools: $J_{21}$ (oxidative carbon transfer from $P_{2}$ to $P_{1}$), $J_{12}$ (cataplerotic redistribution from $P_{1}$ to $P_{2}$), $J_{13}$ (cataplerotic outflow from $P_{1}$ to $P_{3}$), and J_32_ (anaplerotic GABA shunt flux from $P_{3}$ to $P_{2}$). The red-shaded regions indicate the mitochondrial oxidative (Mito_Ox_) phase, and the blue-shaded regions mark the mitochondrial cataplerotic (Mito_Cat_) phase. Initial values are: $P_{1}\left(0\right)=0.4$, $P_{2}\left(0\right)=0.6$, $P_{3}\left(0\right)=0.5$.

The model equations (Equations (1)–(13)) were used to simulate the temporal evolution of metabolite concentrations across all four metabolic pools ($P_{0}$–$P_{3}$). Importantly, the temporal profiles of Ca^2+^ and ATP were not generated by the model but were prescribed as external, experimentally motivated input signals that act as key regulatory drivers of the simulated metabolic dynamics. For clarity, the time axis in Figure 4 is aligned such that time zero corresponds to the onset of the Ca^2+^ pulse.

Figure 4A presents the imposed oscillatory profiles of Ca^2+^ and ATP, while Figure 4B,C show the corresponding dynamics of the metabolite pools and fluxes. The Ca^2+^ pulse initiates the oscillatory sequence by triggering a depletion of the left-side TCA intermediates in pool $P_{2}$. This depletion results from an increase in the flux $J_{21}$ (Figure 4C), which transfers carbon from $P_{2}$ to the right-side TCA pool $P_{1}$. Simultaneously, Pyr derived from the glycolytic pool $P_{0}$ enters the TCA cycle via PDH, contributing to the flux $J_{01}$. In our model, $J_{01}$ is set equal to $J_{21}$, corresponding to the entry of acetyl-CoA into the TCA cycle. However, because Pyr entering via PDH is fully oxidized, it does not contribute to the net replenishment of TCA intermediates in $P_{1}$. Consequently, this phase—driven by Ca^2+^-stimulated PDH activation—results in a net shift of carbon from $P_{2}$ to $P_{1}$ and marks the onset of the mitochondrial oxidative (Mito_Ox_) phase, highlighted in red in Figure 4C.

Shortly thereafter, the GABA pool ($P_{3}$) becomes active, as seen in the declining $P_{3}$ trajectory in Figure 4B. This anaplerotic contribution via the GABA shunt replenishes $P_{2}$ through flux $J_{32}$ (Figure 4C), compensating for its earlier depletion. Because $P_{2}$ was initially emptied into $P_{1}$, it is now capable of accepting fresh carbon from $P_{3}$. This transfer is crucial for maintaining TCA cycling and OxPhos. As a result, $P_{1}$ continues to accumulate while $P_{3}$ declines. This orchestrated sequence—$P_{2}$ → $P_{1}$, then $P_{3}$ → $P_{2}$—builds a TCA cycle rich in carbon intermediates, sustaining NADH/FADH_2_ production and oxidative metabolism during the Mito_Ox_ phase.

As ATP levels rise, the system transitions into the mitochondrial cataplerotic phase (Mito_Cat_), indicated by the blue-shaded region in Figure 4C. In this phase, the TCA cycle—now saturated with carbon—shifts toward net efflux. UCP2 channels facilitate the export of C_4_ dicarboxylates such as OAA, Asp, and Mal, while Cit and αKG are exchanged for Mal in the cytosol. These fluxes promote the production of cytosolic Glu (see Figure 1, inset) and contribute to carbon redistribution from $P_{1}$ back to $P_{2}$ and $P_{3}$, as reflected in rising $J_{12}$ and $J_{13}$. As a consequence, $P_{2}$ and $P_{3}$ refill, while $P_{1}$ becomes depleted (Figure 4B), completing the transition from oxidative to cataplerotic metabolism.

During the final stage of the Mito_Cat_ phase, the PEP cycle becomes fully active. This enables localized ATP production near the plasma membrane, particularly in microdomains adjacent to K_ATP_ channels. The locally elevated ATP concentration promotes K_ATP_ channel closure and initiates the next Ca^2+^ pulse, thereby restarting the cycle with a new Mito_Ox_ phase.

To understand the coupling between glycolysis and TCA dynamics, it is crucial to recognize the central role of Ca^2+^. On one hand, Ca^2+^ pulses are triggered by ATP via K_ATP_ channels, thereby reflecting the redox state of the cell—particularly the metabolic activity within the TCA cycle. On the other hand, the sequestration of Ca^2+^ activates the TCA cycle, enhancing ATP production. This reciprocal Ca^2+^–TCA relationship—where Ca^2+^ both responds to and regulates mitochondrial metabolism—is tightly coupled to glycolysis. Specifically, Ca^2+^ directly influences the glycolytic–mitochondrial interface by promoting Pyr entry into the TCA cycle via PDH activation. This dual regulatory role of Ca^2+^ has been emphasized in previous modeling studies, particularly in the evaluation of the IOM [30]. Notably, Bertram et al. (2023) [30] highlighted that the decline in FBP during the active (oxidative) phase results from elevated Ca^2+^ levels activating PDH, thereby accelerating the conversion of FBP-derived carbon into mitochondrial metabolism. This mechanistic coupling helps explain the experimentally observed “sawtooth” waveform of FBP [3].

**Figure A1:**
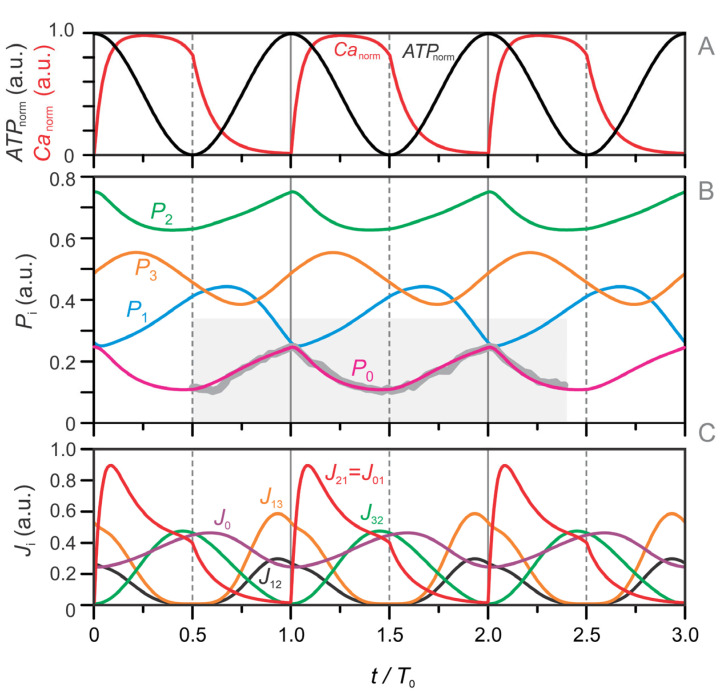
Simulated dynamics of metabolic pools ($P_{0}$–$P_{3}$) and the corresponding fluxes between them. (**A**) Time courses of intracellular Ca^2+^ (red) and ATP (black), which serve as inputs to the model and are fitted to experimental data (see Figure 2 and Equations (1) and (2)). (**B**) Temporal dynamics of the four metabolic pools: the glycolytic pool ($P_{0}$), right-hand TCA cycle intermediates ($P_{1}$), left-hand TCA cycle intermediates ($P_{2}$), and the GABA pool ($P_{3}$). Experimental FBP measurements from Merrins et al. (2016) [3] are shown as a dark gray trace, while the light gray shaded background highlights the corresponding experimental time window, facilitating direct comparison between the model-predicted $P_{0}$ and the experimental observations. (**C**) Key fluxes between pools: $J_{01}$ (flux from $P_{0}$ through PDH), $J_{21}$ (oxidative carbon transfer from $P_{2}$ to $P_{1}$), $J_{12}$ (cataplerotic redistribution from $P_{1}$ to $P_{2}$), $J_{13}$ (cataplerotic outflow from $P_{1}$ to $P_{3}$), and $J_{32}$ (anaplerotic GABA shunt flux from $P_{3}$ to $P_{2}$). Initial values for (**B**,**C**) are: $P_{0}\left(0\right)=0.2$, $P_{1}\left(0\right)=0.4$, $P_{2}\left(0\right)=0.6$, $P_{3}\left(0\right)=0.5$.

In our simplified model, we clearly emphasize the Ca^2+^-dependent flux $J_{01}$, consistent with the findings of Bertram et al. (2023) [30]; however, we do not reproduce the characteristic sawtooth-shaped profile of FBP, as its dynamics are approximated by a sinusoidal function (Equation (5)). To capture the detailed waveform of FBP, the FBP pool ($P_{0}$) would need to be modeled explicitly using a differential equation. This would require not only the efflux term $J_{01}$, but also an influx term $J_{0}$ representing the conversion from F6P to FBP. In Appendix A.1., we demonstrate how such an extension can be implemented to reproduce the sawtooth dynamics. To this purpose, we propose a mechanistically informed formulation of $J_{0}$, which incorporates the well-known ATP and Cit inhibition of PFK1 [31] and the positive feedback loop in which FBP enhances its own production by stimulating PFK1 activity [32,33,34]. As shown in Figure A1, this extended model shows good agreement with the experimentally observed sawtooth-shaped FBP oscillations [3].

To address the functional contribution of the GABA shunt to oscillatory metabolic dynamics, we performed a perturbation analysis focusing on parameter $k_{32}$, which controls the magnitude of the GABA shunt flux $J_{32}$. This analysis examines how changes in GABA-mediated anaplerotic input influence the distribution and amplitude of metabolite oscillations across the modeled pools, without altering the externally imposed ${ATP}_{norm}$ and ${Ca}_{norm}$ phase structure. The results show that modulation of $k_{32}$ primarily affects metabolite amplitudes and pool occupancy, while preserving phase relationships between oscillatory components. Across the explored parameter range, the model exhibited bounded, well-behaved solutions with no drift, divergence, or loss of dynamical consistency, indicating that the DAM framework is numerically stable and structurally robust with respect to variations in GABA shunt activity. Detailed results of this perturbation analysis, including limiting cases, are provided in Appendix A.2.

Because the DAM framework is driven by experimentally prescribed ATP and Ca^2+^ dynamics, an important question is how sensitive the predicted redistribution of metabolic pools is to deviations from the assumed coupling between energetic and redox states. In the core formulation, ATP and NADH are taken to be phase-aligned, reflecting their tight coupling via oxidative phosphorylation. To assess the robustness of this assumption, we performed a dedicated phase-shift analysis in which the normalized redox signal ${NADH}_{norm}$ is progressively shifted in phase relative to ${ATP}_{norm}$. As detailed in Appendix A.3, this analysis shows that redox-regulated fluxes $\left(J_{12},J_{13},J_{32}\right)$ transmit the imposed phase offset directly to mitochondrial and GABA-associated pools, leading to pronounced and pool-specific changes in oscillation amplitudes and phase positions. In particular, a modest phase lead of ${NADH}_{norm}$ relative to ${ATP}_{norm}$ selectively amplifies oscillations in the citrate-dominated pool $P_{1}$, arising from temporal decoupling between oxidative inflow and cataplerotic outflow. These results demonstrate that coordinated anaplerotic–cataplerotic cycling is robust to small redox–energy phase mismatches, while generating specific, testable predictions regarding how redox–energy coordination shapes TCA metabolite dynamics.

In addition, we examined how deviations from the experimentally observed anti-phasic relationship between ATP and Ca^2+^ affect oscillatory carbon redistribution. While the baseline model assumes a dominant anti-phase alignment consistent with beta cell recordings, experimental data indicate that modest phase deviations can occur. Appendix A.4. presents a systematic phase-sensitivity analysis in which the timing of the ATP maximum is shifted relative to the onset of the Ca^2+^ pulse. This analysis shows that altering the ATP–Ca^2+^ phase relationship reshapes the temporal overlap of key inflow and outflow fluxes, thereby modulating oscillation amplitudes in pools $P_{1}$, $P_{2}$, and $P_{3}$, while preserving oscillatory structure over a broad parameter range. In particular, conditions in which ATP dynamics precede Ca^2+^ activation ($\tau_{ATP}>0$) are predicted to enhance oscillatory excursions of the citrate-dominated pool $P_{1}$. These findings constitute falsifiable predictions of the DAM framework, highlighting the role of energetic–Ca^2+^ phase coordination as a key constraint shaping oscillatory metabolic redistribution under experimentally observed input dynamics.

### 3.1. Stock–Flow Diagrams Illustrating Model Behavior

**Figure 5:**
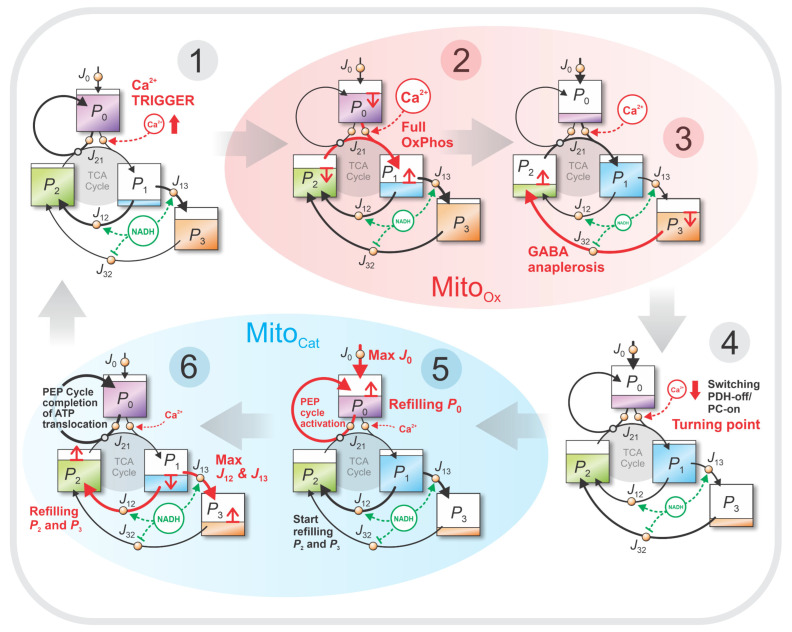
Stock–flow diagrams illustrating the stepwise progression of the model’s metabolic oscillation cycle. Six characteristic stages are shown. Steps 2 and 3 represent the mitochondrial oxidative (Mito_Ox_) phase (red shaded), characterized by oxidative carbon flux and active NADH production. Steps 5 and 6 correspond to the mitochondrial cataplerotic (Mito_Cat_) phase (blue shaded). Steps 1 and 4 are transitional: Step 1 depicts Ca^2+^-induced activation of PDH and entry into the Mito_Ox_ phase, while Step 4 reflects the turning phase from Mito_Ox_ to Mito_Cat_, characterized by the main switch PDH-off/PC-on. Arrow widths qualitatively indicate flux magnitude, and shading levels within each pool reflects simulated metabolite concentrations (corresponding to the values in Figure 4B). Note that $J_{12}$ is not part of the TCA cycle (see Equation (9)); the TCA cycle itself is shown as gray-shaded circle. Metabolite levels in pools $P_{0}$, $P_{1}$, $P_{2}$, and $P_{3}$ are shown relative to their respective minimum and maximum values, so that all pools can be compared on the same scale of filling and emptying, while their absolute oscillation spans differ: the largest amplitude occurs in $P_{1}$ ($\Delta P_{1}=0.22$), followed by $P_{0}$ ($\Delta P_{0}=0.20$), $P_{3}$ ($\Delta P_{3}=0.18$), and $P_{2}$ ($\Delta P_{2}=0.16$).

To enhance intuitive understanding of the model’s dynamic behavior, we present a sequence of stock–flow diagrams (Figure 5) that illustrate the stepwise filling and emptying of the four metabolic pools ($P_{0}$–$P_{3}$) throughout the oscillatory cycle. These diagrams highlight the system’s progression through key transitional states and emphasize how metabolite pool levels and inter-pool fluxes evolve during the Mito_Ox_ and Mito_Cat_ phases.

In this representation, stocks refer to the concentrations of the metabolic pools ($P_{0}$–$P_{3}$), while flows denote the key metabolic fluxes: $J_{01}$, $J_{12}$, $J_{13}$, $J_{21}$, and $J_{32}$. The glycolytic inflow $J_{0}$ is also shown for completeness, even though it is not explicitly defined in the core model. However, $J_{0}$ is analyzed in more detail in the Appendix A.1.

Figure 5 illustrates six characteristic stages of the metabolic oscillation cycle:

Steps 2 and 3 represent the mitochondrial oxidative (Mito_Ox_) phase (shaded red).

Steps 5 and 6 represent the mitochondrial cataplerotic (Mito_Cat_) phase (shaded blue).

Steps 1 and 4 correspond to transitional phases that initiate/terminate the core oscillatory phases.

Step 1—Ca^2+^-Triggered Entry into Mito_Ox_ Phase 

This initial stage begins with a Ca^2+^ pulse that activates PDH, opening flux $J_{01}$ and allowing fresh carbon from the glycolytic pool $P_{0}$ to enter the TCA cycle. At this point, $P_{1}$ is relatively empty, primed to receive carbon from $P_{2}$ and $P_{0}$. Fluxes $J_{21}$ and $J_{01}$ begin to rise in parallel, initiating the oxidative cycle.

Step 2—Maximal OxPhos Phase

With strong PDH activation, high fluxes through $J_{01}$ and $J_{21}$ (highlighted in red) are initiated, leading to a temporary depletion of $P_{2}$. Note that $J_{21}=J_{01}$, because Cit formation in $P_{1}$ requires an equal input of Pyr (from $P_{0}$) and Mal-derived OAA (from $P_{2}$). These high fluxes support NADH and FADH_2_ production and active OxPhos. During this stage, the TCA cycle operates fully in the clockwise direction (gray-shaded cycle in Figure 5), although the arrows are not drawn for the entire cycle. To avoid confusion with the graphical presentation, note that flux $J_{12}$ is not part of the TCA cycle (see Equation (9)); its arrow is shown outside the gray-shaded cycle.

Step 3—GABA Shunt-Driven Anaplerosis

Following the temporary depletion of $P_{2}$, the GABA shunt (via $J_{32}$, shown in red) replenishes $P_{2}$, allowing TCA cycling to continue. This carbon input from $P_{3}$ sustains the Mito_Ox_ phase by restoring balance between the left and right TCA segments ($P_{2}$ and $P_{1}$) and by providing additional NADH and FADH_2_ through GABA-derived Succ entering the TCA cycle.

Step 4—Transition to Mito_Cat_ Phase

This stage represents the metabolic turning point. Rising ATP levels and accumulation of TCA intermediates, especially in $P_{1}$, cause oxidative cycling to slow and begin reversing. This initiates cataplerosis—extrusion of TCA intermediates—together with cataplerotic refilling of $P_{2}$ and $P_{3}$. As regulatory signals shift (including declining Ca^2+^ together, changes in NADH/NAD^+^, and other metabolites), glycolytic influx is redirected from PDH toward PC. This PDH-off/PC-on switch, under conditions of high GTP, activates the PEP cycle.

Step 5—Growing Cataplerosis, PEP Cycling, and $P_{0}$ Refilling

Cataplerotic fluxes intensify, with carbon extruded from $P_{1}$ to $P_{2}$ and $P_{3}$. At high GTP levels, and with $P_{0}$ rising ($J_{0}$ reaching its maximum at this stage), fluxes through the PEP cycle increase, efficiently translocating ATP from mitochondria into cytosolic microdomains near K_ATP_ channels.

Step 6—Cataplerotic Refilling of $P_{2}$ and $P_{3}$

Cataplerosis now reaches its full extent. As GTP declines (consumed by the active PEP cycle), UCP2 inhibition weakens, enabling strong extrusion of C_4_ units—mainly Asp and Mal. Together with Cit–Mal shuttling, this drives redistribution of intermediates through cataplerotic pathways converting αKG into Glu and further into GABA (see inset of Figure 1). Carbon is efficiently redistributed from $P_{1}$ to both $P_{2}$ and $P_{3}$ via large fluxes $J_{12}$ and $J_{13}$ (red arrows outside the gray-shaded TCA cycle). This final stage prepares the system for the next oscillation: $P_{0}$ and $P_{2}$ are fully replenished, while $P_{3}$ is in its final refilling phase. At the same time, the still-active PEP cycle completes ATP translocation to microdomains near K_ATP_ channels, setting the conditions for K_ATP_ channel closure and the next Ca^2+^ pulse.

### 3.2. Simulated Animation of Metabolic Pool Dynamics

**Figure 6:**
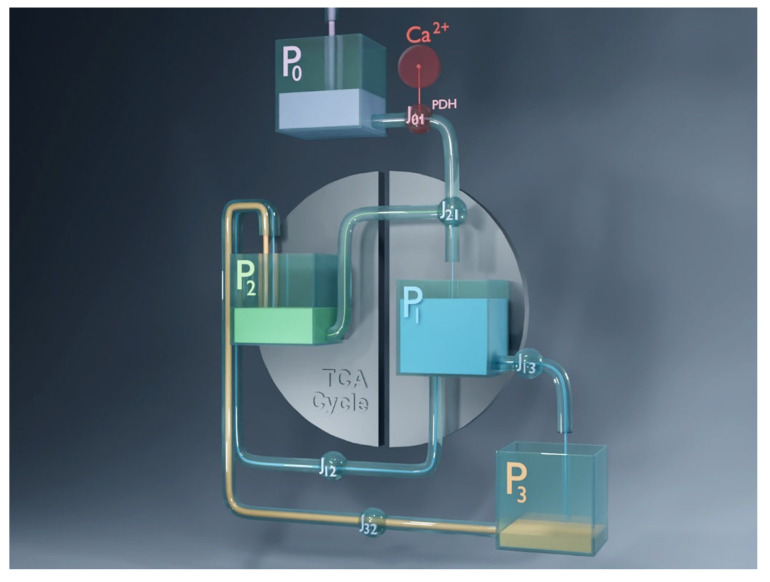
Snapshot from the animated visualization of the four-pool model dynamics. The four metabolic pools ($P_{0}$–$P_{3}$) are connected by pipelines representing the fluxes between them as flowing liquid. The animation, created in *Blender*, is based on the numerically integrated model in its most detailed form, as described in Appendix A.2: $P_{0}$ dynamics is governed by differential Equation (A1), and $J_{0}$ regulation is implemented by Equation (A2). It depicts the cyclic emptying and refilling of pools during glucose-stimulated oscillations in beta cells and highlights the transitions between the Mito_Ox_ and Mito_Cat_ phases. The full animation is available at: https://doi.org/10.5281/zenodo.16951481.

To better illustrate the model’s oscillatory behavior, we created an animated visualization showing the cyclic emptying and refilling of the four metabolic pools. The animation was generated in Blender and depicts the pools connected by pipelines, with fluxes represented as flowing liquid. It is based directly on the numerically simulated dynamics of the model in its most detailed form: the core system of equations described in the Model section, with the dynamics of $P_{0}$ defined explicitly and the $J_{0}$ flux modeled with ATP-, FBP-, and Cit-dependent regulation as in Appendix A.2. This ensures that the animation faithfully reflects the simulated metabolite and flux oscillations. A snapshot from the animation is shown in Figure 6, and the full video is available at: https://doi.org/10.5281/zenodo.16951481.

## 4. Discussion

In this study, we employed a deliberately simplified, phenomenological computational framework to examine how the GABA shunt can be functionally integrated into established metabolic mechanisms of pancreatic beta cell insulin secretion. Rather than attempting to generate oscillations autonomously, the model was designed to investigate how experimentally observed ATP/ADP and Ca^2+^ dynamics constrain metabolic flux partitioning within a coupled anaplerotic–cataplerotic system. Within this scope, DAM does not serve as a generator of oscillations, but as a structured mapping between imposed energetic and Ca^2+^ signals and the resulting redistribution of carbon among interacting metabolic modules.

A central and model-derived outcome of this analysis is that carbon entry via the GABA shunt—through succinate into the TCA cycle—cannot be treated as an isolated anaplerotic contribution. Once explicitly represented as a dynamic metabolite pool (*P*_3_), GABA-derived carbon necessarily engages a set of closure constraints that follow directly from mass balance and flux continuity. In particular, effective utilization of GABA shunt-mediated anaplerosis requires coordinated cataplerotic export from the TCA cycle, primarily involving α-ketoglutarate, which in turn depends on obligatory counter-fluxes of C_4_ metabolites such as malate and aspartate. In the DAM framework, this cataplerotic exchange is balanced by complementary anaplerotic replenishment via pyruvate carboxylase (PC). Importantly, this interdependence is not imposed heuristically but emerges as an inevitable structural requirement once GABA metabolism is introduced explicitly and coupled to TCA cycling.

This explicit representation of a GABA-associated metabolic pool distinguishes DAM from existing modeling frameworks that describe beta cell metabolism without resolving GABA dynamics as an independent, time-evolving reservoir. By introducing *P*_3_ and its associated fluxes, DAM uniquely enables analysis of how GABA production, depletion, and replenishment interact dynamically with TCA intermediates and PC-mediated anaplerosis under phase-locked energetic and Ca^2+^ inputs. As a consequence, the model generates nontrivial predictions regarding the timing, amplitude, and phase relationships of citrate-, glutamate-, and GABA-related fluxes—quantities that are not prescribed by experimental inputs but arise as solutions of the coupled equations.

An important implication of this organization is that anaplerotic influx through PC and the GABA shunt is closely counterbalanced by cataplerotic outflows, rendering the system effectively quasi-closed and governed primarily by internal flux redistribution among $P_{1}$, $P_{2}$, and $P_{3}$ pools. Notably, the net anaplerotic fluxes required to sustain this cycling remain modest, consistent with experimental evidence indicating that more than 80% of glucose taken up by beta cells is fully oxidized [35]. This observation supports the view that beta cell metabolism is optimized for signaling rather than for net biomass accumulation, in line with their specialized endocrine function. Accordingly, anaplerotic–cataplerotic cycling in beta cells is best interpreted as serving regulatory and signaling functioning roles rather than primarily biosynthetic needs [36]. Nevertheless, biosynthetic pathways—particularly fatty-acid synthesis—remain functionally important and can be tightly coupled to metabolic signaling. For example, short-chain acyl-CoAs generated from Cit exported cataplerotically from mitochondria exert potent regulatory effects on beta cell function, especially in rodent islets [37]. More recent studies further suggest that fatty-acid biosynthesis may contribute to GSIS by replenishing membrane lipids required for vesicle-mediated exocytosis and/or by providing an electron sink to accommodate increased glucose catabolism [38]. In addition, the biosynthetic fluxes appear particularly relevant during beta cell development, as indicated by enhanced reductive TCA cycle activity observed in human pluripotent stem cell-derived islets [39]. Together, these findings underscore the importance of considering both oxidative and reductive TCA pathways and provide strong conceptual support for modeling frameworks in which metabolic flexibility emerges from coordinated flux partitioning—an organizational principle that is made explicit and quantitatively tractable within the DAM framework.

The dynamics of TCA metabolites—and their fluctuations in the cytosol via cataplerotic export—have been measured with high temporal resolution, allowing direct comparison with our model predictions. A particularly compelling line of evidence comes from MacDonald et al. (2003) [40], who measured mitochondrial Cit oscillations and identified Cit as the most prominently oscillating TCA intermediate. Consistently, our simulations predict that Cit oscillations are the most pronounced among TCA metabolites: the $P_{1}$ pool is emptied during the Mito_Ox_ phase (via fluxes from $P_{2}$ and $P_{0}$), replenished via $P_{2}$ and the GABA shunt, and then depleted again during Mito_Cat_. The timing of maximal cataplerotic flux ($J_{12}$) in our model coincides with the experimentally observed anti-phasic oscillations of cytosolic Cit reported by Gregg et al. (2019) [19]. Furthermore, MacDonald et al. (2003) [40] emphasized Cit’s function as a potent PFK inhibitor, capable of modulating glycolytic flux and synchronizing mitochondrial activity. This feedback role of Cit strongly supports our concept of a synergistic, integrated metabolic network in which glycolysis, the TCA cycle, and the GABA shunt operate in tight coordination. Incorporating this feedback mechanism directly into our model has contributed to the improved predictive accuracy described in Appendix A.1.

Beyond reproducing known oscillatory patterns, DAM generates specific, testable predictions regarding the conditions under which Cit oscillations become dominant. In particular, the model predicts that the citrate-dominated pool $P_{1}$ exhibits selectively enhanced oscillation amplitudes when the redox signal ${NADH}_{norm}$ leads the energetic signal ${ATP}_{norm}$ (i.e., $\tau_{NADH}>0$; Appendix A.3). This behavior arises inevitably from the structure of the model: a redox lead shifts NADH-dependent cataplerotic fluxes ($J_{12},J_{13},J_{32}$) earlier in the cycle relative to oxidative inflow ($J_{21}$), thereby amplifying net accumulation and depletion of $P_{1}$. This constitutes a falsifiable model prediction, linking redox–energy phase coordination to the dominance of citrate-related oscillations observed experimentally in beta cells.

We further examined the sensitivity of metabolic dynamics to deviations from the experimentally observed anti-phase relationship between ATP and Ca^2+^ (Appendix A.4). The analysis shows that increasing the phase offset parameter $\tau_{ATP}$, such that ATP dynamics precede Ca^2+^ activation, leads to a systematic increase in the oscillation amplitude of the citrate-dominated pool $P_{1}$. This again represents a falsifiable prediction of the DAM framework. Specifically, the model raises the testable hypothesis that, under physiological conditions, ATP dynamics may need to precede Ca^2+^ activation by a small but finite interval ($\tau_{ATP}>0$) to sustain robust oscillatory redistribution within TCA-associated pools. Consistent with this possibility, some experimental studies report closer alignment ($\tau_{ATP}\approx 0$) between ATP and Ca^2+^ signals under certain pathophysiological conditions (e.g., ob/ob mouse models), whereas in other cases—predominantly in control conditions—ATP dynamics appear to precede Ca^2+^ pulses [19]. However, this distinction is not uniformly observed across studies, and additional high-resolution experimental data will be required to determine whether this phase relationship constitutes a defining feature of physiological regulation and how it may be altered in different pathological states.

In this context, it would be particularly informative to assess whether the pronounced citrate oscillations reported by MacDonald et al. (2003) [40]—identifying citrate as the most prominently oscillating TCA intermediate—are preferentially associated with conditions in which ATP dynamics precede Ca^2+^ activation ($\tau_{ATP}>0$), and to what extent a redox lead relative to ATP ($\tau_{NADH}>0$) contributes to this behavior. Such experiments would directly test the DAM framework’s predictions regarding the coupling between energetic–Ca^2+^ coordination and TCA metabolite dynamics.

Finally, DAM also generates specific predictions regarding glutamate and GABA dynamics. The model predicts that cytosolic glutamate peaks during the Mito_Cat_ phase, in agreement with experimental observations by Lewandowski et al. (2020) [20] and with the mechanistic conversion of αKG to Glu described by Grubelnik et al. (2024) [7]. Beyond its amplifying role in insulin secretion [7], Glu also contributes to refilling the GABA pool ($P_{3}$) toward the end of the Mito_Cat_ phase. This timing is consistent with the observations of Menegaz et al. (2019) [29], who showed pulsatile GABA dynamics and proposed that GABA contributes both to terminating insulin secretion bursts and to synchronizing the rhythm of pulsatile release. In our model, GABA replenishment at the end of the Mito_Cat_ phase aligns precisely with these experimental findings, linking cataplerotic fluxes to both the termination and resynchronization of GSIS cycles.

To place these results in proper context, we emphasize that the DAM is intentionally constructed as a minimal, phenomenological model. Several dynamic components—most notably Ca^2+^ and ATP/ADP profiles—are prescribed based on experimental observations rather than generated endogenously, and the internal dynamics are simplified to focus primarily on phase relationships and the coordinated filling and emptying of metabolic pools. Consequently, the detailed waveforms of individual metabolites are represented only approximated. For instance, FBP is described as a smooth oscillatory signal in the core model, whereas Appendix A.2. demonstrates that its experimentally observed “sawtooth” profile [3] can be reproduced when additional regulatory detail is introduced into the glycolytic influx $J_{0}$. These considerations underscore that DAM is not intended as a comprehensive or definitive description of beta cell metabolism, but rather as a flexible conceptual scaffold that isolates and clarifies key constraints governing coordinated anaplerotic–cataplerotic dynamics.

A major strength of the DAM framework lies in its deliberately minimal and modular architecture, which enables systematic refinement and targeted extension without altering the core conceptual structure. In particular, the reduction in the TCA cycle into two functionally distinct pools reflects a coarse-grained representation designed to capture the dominant directions of carbon flow and redox-sensitive redistribution rather than detailed enzyme-level kinetics. This partitioning is not arbitrary, but is motivated by well-established functional asymmetries within the TCA cycle and by broader evolutionary considerations, in which the cycle can be viewed as comprising partially decoupled oxidative and reductive segments. Within this abstraction, grouping metabolites into synchronized pools provides a tractable means of studying system-level constraints on carbon redistribution under oscillatory energetic and Ca^2+^ inputs.

At the same time, we emphasize that this pooling assumption represents a modeling choice rather than a biological assertion of perfect synchrony among individual metabolites. Relaxing this assumption by introducing finer-grained compartmentalization or explicitly resolving intra-pool heterogeneity constitutes a natural direction for future studies. Importantly, the present results demonstrate that the central conclusions of DAM—namely the necessity of coordinated anaplerotic input via the GABA shunt and pyruvate carboxylase, balanced by regulated cataplerotic exchange—arise at the level of carbon flow topology and phase coordination, and do not depend on metabolite-level resolution.

Beyond its conceptual role, the DAM framework provides a concrete and flexible platform for exploring physiological regulation of beta cell metabolism, as it allows direct examination of how experimentally observed ATP/ADP and Ca^2+^ dynamics constrain metabolic flux partitioning across coupled anaplerotic and cataplerotic pathways. Within this context, the model enables systematic interrogation of how changes in pathway capacity, transport efficiency, or redox sensitivity reshape the redistribution of carbon among glycolytic, TCA, and GABA-associated pools.

In a pathophysiological context, the same framework can be used to probe specific, experimentally motivated perturbation scenarios—for example altered GABA shunt throughput, modified PC activity, or changes in mitochondrial redox coupling—without presupposing a specific disease mechanism. Such perturbations can be implemented in a controlled manner to assess their consequences for metabolic pool occupancy, flux balance, and phase-dependent coordination, thereby providing testable hypotheses for conditions such as metabolic stress, impaired insulin secretion, or early dysregulation observed in prediabetes and type 2 diabetes.

An additional direction is the explicit coupling of the metabolic core to electrophysiological models of the plasma membrane, thereby linking metabolic flux redistribution to membrane potential dynamics and Ca^2+^ handling. Together, these extensions would enable a more comprehensive investigation of how metabolic, electrical, and Ca^2+^ oscillations are coordinated under physiological conditions and how this coordination may become disrupted in pathophysiological states. In this sense, while DAM is deliberately minimal by design, its modular structure provides a robust foundation for iterative refinement and hypothesis-driven exploration of the metabolic and signaling networks underlying pulsatile insulin secretion.

## Figures and Tables

**Figure A2:**
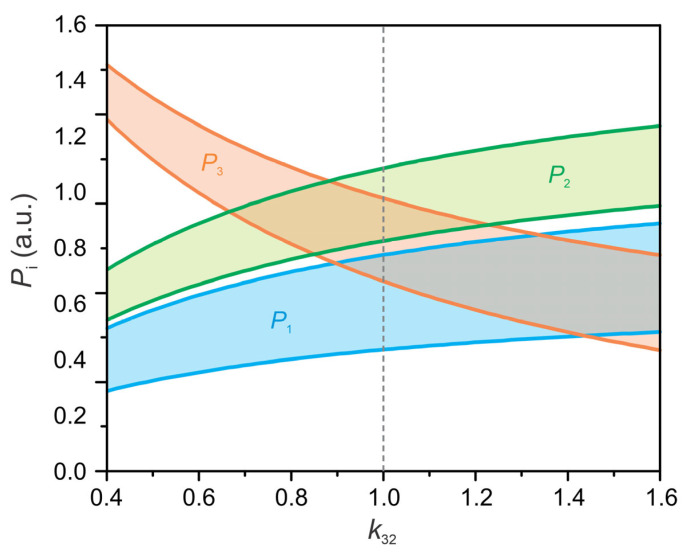
Perturbation analysis of parameter $k_{32}$, which regulates the magnitude of the GABA shunt flux $J_{32}$. Shown are the maximum and minimum oscillatory values of metabolite concentrations in pools $P_{1}$, $P_{2}$, and $P_{3}$ as a function of $k_{32}$. Variation of $k_{32}$ alters the relative distribution and amplitude of metabolite concentrations across pools, while phase relationships between oscillations remain unchanged. The dashed line indicates the reference value $k_{32}=1$, which was used in all baseline simulations (Equation (12)).

**Figure A3:**
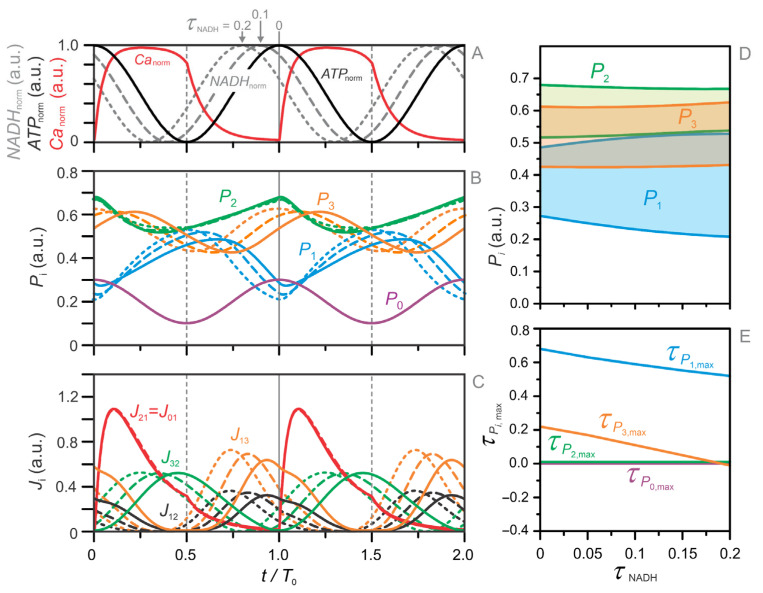
Analysis of the effect of a phase lead of the ${NADH}_{norm}$ signal relative to ${ATP}_{norm}$. Results shown with long dashed lines correspond to $\tau_{NADH}=0.1$, and short dashed lines to $\tau_{NADH}=0.2$; this line-style convention applies to panels (**A**–**C**). (**A**) Time courses of ${Ca}_{norm}$ (red), ${ATP}_{norm}$ (black), and the phase-shifted ${NADH}_{norm}$ (gray). (**B**) Temporal dynamics of the metabolic pools $P_{0}$–$P_{3}$. (**C**) Corresponding fluxes between pools. (**D**) Oscillation ranges of metabolite concentrations in pools $P_{1}$, $P_{2}$, and $P_{3}$ as a function of $\tau_{NADH}$. (**E**) Phase offsets $\tau_{P_{i,max}}$ indicating the time within the oscillation period at which each pool reaches its maximal value, shown as a function of $\tau_{NADH}$.

**Figure A4:**
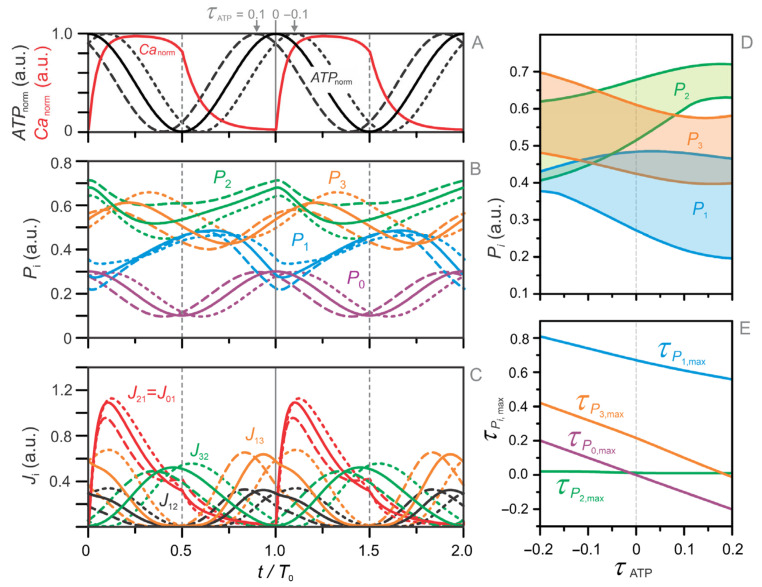
Analysis of the phase shift in the ATP signal maximum relative to the onset of the Ca^2+^ pulse. Results shown with long dashed lines correspond to a phase lead of $\tau_{ATP}=0.1$, whereas short dashed lines correspond to a phase lag of $\tau_{ATP}=-0.1$; this line-style convention applies to panels (**A**–**C**). (**A**) Time courses of ${Ca}_{norm}$ (red) and ${ATP}_{norm}$ (black). (**B**) Temporal dynamics of the metabolic pools $P_{0}$–$P_{3}$. (**C**) Corresponding fluxes between pools. (**D**) Oscillation ranges of metabolite concentrations in pools $P_{1}$, $P_{2}$, and $P_{3}$ as a function of $\tau_{ATP}$. (**E**) Phase offsets $\tau_{P_{i,max}}$, defined as the time within the oscillation period at which each pool reaches its maximal value, shown as a function of $\tau_{ATP}$.

## Data Availability

The original contributions presented in this study are included in the article. Further inquiries can be directed to the corresponding author.

## Abbreviations

The following abbreviations are used in this manuscript:

AcCoA	acetyl-coenzyme A
AGC	aspartate-glutamate carrier
αKG	α-ketoglutarate
αKGDH	α-KG dehydrogenase
Asp	Aspartate
ATP	adenosine triphosphate
ATP_norm_	normalized ATP signal
Ca_norm_	normalized Ca^2+^ signal
CIC	citrate-isocitrate carrier
Cit	citrate
DAM	dual anaplerotic model
DOM	dual oscillator model
FADH_2_	flavin adenine dinucleotide
FBP	fructose-1,6-bisphosphate
Fum	fumarate
GABA	gamma-aminobutyric acid
GABA-TK	GABA transaminase
GAD	glutamate decarboxylase
Glu	glutamate
Gln	glutamine
GLS	glutaminase
GOT1	glutamate-oxaloacetate transaminase 1
GOT2	glutamate-oxaloacetate transaminase 2
GSIS	glucose-stimulated insulin secretion
GTP	guanosine-triphosphate
H_2_O_2_	hydrogen peroxide
IDH	isocitrate dehydrogenase
IOM	integrated oscillator model
K_ATP_	ATP-sensitive potassium
Mal	malate
MDH	malate dehydrogenase
ME1	malic enzyme
NADH	nicotinamide adenine dinucleotide
NADH_norm_	normalized NADH signal
NADPH	nicotinamide adenine dinucleotide phosphate
OAA	oxaloacetate
OGC	oxoglutarate carrier
OxPhos	oxidative phosphorylation
PC	pyruvate carboxylase
PCK2	phosphoenolpyruvate carboxykinase (mitochondrial isoform)
PEP	phosphoenolpyruvate
PDH	pyruvate dehydrogenase
PFK	phosphofructokinase
PK	pyruvate kinase
Pyr	pyruvate
ROS	reactive oxygen species
SCS	succinyl-CoA synthetase
SSADH	succinic semialdehyde dehydrogenase
Succ	succinate
SuCoA	succinyl-CoA
TCA	tricarboxylic acid
UCP2	uncoupling protein 2

## Appendix A

### Appendix A.1. Modeling $J_{0}$ with ATP, FBP, and Cit Regulation

In the core version of the model, glycolytic input was implicitly linked to the experimentally prescribed ATP dynamics. However, once the glycolytic influx $J_{0}$ is introduced explicitly as a flux term, the dynamics of the corresponding metabolite pool $P_{0}$ must also be described explicitly. This extension allows glycolytic substrate availability to respond dynamically to regulatory signals while remaining consistent with the phenomenological structure of the model. Accordingly, the temporal evolution of $P_{0}$ is described by the following differential equation:

(A1) $$\frac{{dP}_{0}}{dt}=J_{0}-J_{01},$$

where $J_{01}$ represents the downstream consumption of metabolites from pool $P_{0}$. The flux $J_{0}$ incorporates established inhibitory and activating effects on PFK1 and is modeled as:

(A2) $$J_{0}=k_{1}P_{0}^{n_{0}}P_{1}(ATP_{0}-ATP)+k_{0},$$

where $k_{1}=1.5$, $k_{0}=0.2$, $ATP_{0}=1.2$, and $n_{0}=0.5$. The first term represents the well-known inhibition of PFK1 by ATP [21,31], modeled through the difference $\left(ATP_{0}-ATP\right)$, such that a drop in ATP—during Ca^2+^-stimulated ATPase activity—leads to an increase in $J_{0}$. The second component captures the positive feedback of FBP on its own production via PFK1 activation ($P_{0}^{n_{0}}$), as established in earlier studies [4,32,33,34], with $n_{0}=0.5$ chosen to emphasize this effect relative to the FBP level. Finally, Cit inhibition of PFK1 is incorporated through the $P_{1}$ term, where $P_{1}$ represents mitochondrial Cit, which oscillates in antiphase to cytosolic Cit [19,40], making it a suitable proxy for cytosolic Cit inhibition. The constants $k_{0}$ and $k_{1}$ were selected to ensure that, for the oscillating values of ATP, $P_{0}$, and $P_{1}$, the flux $J_{0}$ provides sufficient substrate input to maintain metabolite oscillations within the physiological range.

The simulated dynamics of metabolic pools ($P_{0}$–$P_{3}$) and the corresponding fluxes between them are shown in Figure A1. When cytosolic Ca^2+^ rises, ATP begins to decline due to ATPase activity, which stimulates PFK via the ATP-dependent term and causes $J_{0}$ to increase, despite the concurrent decline in FBP ($P_{0}$) and the resulting loss of its positive feedback on PFK1 activation. Meanwhile, cytosolic Cit drops, and at the midpoint of the Ca^2+^ pulse—when ATP reaches its minimum—$J_{0}$ continues to rise slightly due to the reduced cytosolic Cit (reflected in the increase in $P_{1}$). Toward the end of the pulse, ATP and cytosolic Cit recover, and $J_{0}$ decreases, although elevated $P_{0}$ sustains it above baseline. Notably, the model reproduces oscillations of $P_{0}$ with the characteristic “sawtooth-like” profile observed experimentally for FBP oscillations in beta cells [3].

### Appendix A.2. Perturbation Analysis of GABA Shunt-Mediated Anaplerosis

To assess the functional role of the GABA shunt within DAM, we performed a perturbation analysis focusing on parameter $k_{32}$, which regulates the magnitude of the GABA shunt flux $J_{32}$. This analysis examines how modulation of GABA-mediated anaplerotic input affects the distribution and oscillatory amplitudes of metabolite pools while preserving the externally imposed phase structure.

We systematically varied parameter $k_{32}$ over a physiologically plausible range and quantified the resulting changes in metabolite dynamics. Altering $k_{32}$ primarily affects the relative amplitudes and mean levels of metabolite concentrations in pools $P_{1}$, $P_{2}$, and $P_{3}$. Importantly, although the absolute and relative concentrations shift in response to changes in GABA shunt activity, the phase relationships between oscillations in the different pools remain unchanged. This indicates that, within the DAM framework, the GABA shunt modulates carbon partitioning among metabolic pools rather than controlling the timing of oscillatory transitions.

Figure A2 summarizes this perturbation analysis by showing the maximum and minimum values of oscillatory metabolite concentrations in pools $P_{1}$, $P_{2}$, and $P_{3}$ as a function of $k_{32}$. These results demonstrate that the GABA shunt acts as a key regulator of oscillatory amplitude and pool occupancy, while the overall phase structure of the system is preserved.

### Appendix A.3. Phase Shift Between NADH and ATP

In the core formulation of the model, the energetic state of the cell (ATP) and its redox state (NADH) are assumed to be phase-aligned, such that the normalized redox proxy is defined as ${NADH}_{norm}(t)\equiv {ATP}_{norm}(t)$ (Equation (4)). This assumption reflects the strong coupling between mitochondrial redox balance and ATP synthesis via oxidative phosphorylation. Nevertheless, in pancreatic beta cells a finite phase offset between NADH and ATP oscillations cannot be excluded. Such a delay is biologically plausible, as changes in the redox state precede ATP synthesis in the mitochondrial respiratory chain, implying a causal ordering in which NADH dynamics may lead ATP dynamics.

To assess the sensitivity of the DAM framework to a possible mismatch between redox and energetic signaling, we performed a perturbation analysis introducing a constant phase shift between the normalized NADH and ATP signals. Specifically, we define the redox proxy as

(A3) $${NADH}_{\mathrm{norm}}(t/T_{0})={ATP}_{\mathrm{norm}}(t/T_{0}+\tau_{\mathrm{NADH}}),$$

where $\tau_{\mathrm{NADH}}\in [0,1]$ is a dimensionless phase-shift parameter expressed as a fraction of the oscillation period $T_{0}$. A value of $\tau_{NADH}=1$ corresponds to a full-period phase shift.

Figure A3 summarizes the effects of progressively increasing $\tau_{NADH}$ on the system dynamics. Panel A illustrates the imposed phase divergence between the energetic signal $ATP_{norm}$, the redox proxy $NADH_{norm}$, and the normalized calcium signal $Ca_{norm}$. Panel B shows the resulting temporal evolution of the metabolic pools $P_{i}$. As expected, the dynamics of the glycolytic pool $P_{0}$ remains unchanged, because $P_{0}$ is directly linked to ATP oscillations (Equation (5)) and therefore remains phase-aligned with $ATP_{norm}$ (see Figure 3). In contrast, the mitochondrial and GABA-related pools $P_{1}$, $P_{2}$, and $P_{3}$ exhibit pronounced changes in both oscillation amplitude and phase position within the period, with pool-specific sensitivity to $\tau_{NADH}$. Panel C shows the corresponding metabolic fluxes. Fluxes that explicitly depend on $NADH_{norm}$—namely $J_{12}$, $J_{13}$, and $J_{32}$—are phase-shifted in direct accordance with the imposed $\tau_{NADH}$. When $NADH_{norm}$ leads $ATP_{norm}$, this induces a temporal mismatch between the oxidative inflow into pool $P_{1}$ (primarily via $J_{21}$) and its redox-regulated cataplerotic outflows ($J_{12}$ and $J_{13}$), thereby promoting transient accumulation and depletion within $P_{1}$.

These effects are quantified in panel D, which displays the oscillation ranges of pools $P_{1}$, $P_{2}$, and $P_{3}$—defined as the difference between maximal and minimal values within one oscillation period—as a function of $\tau_{NADH}$. With increasing $\tau_{NADH}$, the oscillation amplitude of $P_{1}$ increases monotonically and becomes markedly larger than those of $P_{2}$ and $P_{3}$, indicating a selective amplification of the citrate-dominated pool in response to redox–energy phase mismatch. Panel E shows the phase positions $\tau_{P_{i,max}}$ at which each pool reaches its maximal value, illustrating how a redox lead propagates into the phase organization of metabolic reservoirs. Together, these results demonstrate that a modest phase lead of NADH relative to ATP is sufficient to selectively enhance oscillatory excursions of $P_{1}$, providing a clear, testable model prediction linking redox–energy coordination to the dominance of citrate-related oscillations observed experimentally.

### Appendix A.4. Phase Relationship Between Ca^2+^ and ATP Oscillations

Based on experimental observations (see Figure 2), the baseline formulation of the model assumes an anti-phasic relationship between ATP and cytosolic Ca^2+^, with ATP reaching its maximum when Ca^2+^ concentration is minimal—typically just prior to the onset of the Ca^2+^ pulse. This anti-phase relationship represents the dominant and most reproducible pattern reported in beta-cell experiments and forms the reference state used throughout the main simulations. At the same time, high-resolution experimental recordings indicate that small deviations from perfect anti-phase alignment can occur under certain conditions, with the ATP maximum exhibiting a modest phase offset relative to the Ca^2+^ signal [19]. To assess the sensitivity of the DAM framework to such experimentally plausible deviations, we systematically examined how controlled phase shifts in the energetic state relative to the Ca^2+^ signal affect the oscillatory redistribution of metabolites across the modeled pools.

To this end, we introduce a dimensionless phase-shift parameter $\tau_{ATP}\in [-1,1]$, which defines the temporal shift in the ATP maximum relative to the onset of the Ca^2+^ pulse. Values $\tau_{ATP}=\pm 1$ correspond to shifts in one full oscillation period. In Figure A4, we illustrate two representative cases: (i) a phase lead of ATP relative to Ca^2+^ ($\tau_{ATP}=0.1$), in which ATP reaches its maximum before the Ca^2+^ pulse, and (ii) a phase lag ($\tau_{ATP}=-0.1$), in which the ATP maximum occurs after Ca^2+^ activation. Panel B of Figure A4 shows the resulting dynamics of metabolite concentrations in the individual pools $P_{0}–P_{3}$. As expected, the glycolytic pool $P_{0}$ remains phase-aligned with ATP by construction (Equation (5)). In contrast, the mitochondrial and GABA-related pools exhibit pronounced sensitivity to the ATP–Ca^2+^ phase relationship. Panel C displays the corresponding flux dynamics, demonstrating that shifts in the ATP phase alter both the temporal alignment and relative overlap of key inflow and outflow fluxes.

The quantitative impact of the phase shift is summarized in panel D, which shows the oscillation ranges (defined as maximal minus minimal values within one period) of pools $P_{1}$, $P_{2}$, and $P_{3}$ as a function of $\tau_{ATP}$. With increasing $\tau_{ATP}$, the oscillation amplitude of pool $P_{1}$ increases, while the amplitude of pool $P_{2}$ decreases. This behavior arises primarily from the changing temporal relationship between the inflow into $P_{1}$ (via $J_{21}$) and the outflow mediated by the GABA shunt (via $J_{13}$). When $\tau_{ATP}=-0.1$, these fluxes substantially overlap in time, limiting net accumulation and depletion of $P_{1}$. In contrast, for $\tau_{ATP}=0.1$, their temporal separation allows larger excursions of $P_{1}$, resulting in increased oscillation amplitudes. These results highlight the critical role of phase coordination between energetic and Ca^2+^ signals in shaping GABA shunt-mediated carbon redistribution. Panel E depicts the relative phase offsets $\tau_{P_{i,max}}$, defined as the time within the oscillation period at which each pool reaches its maximal value, measured relative to the onset of the Ca^2+^ pulse. Consistent with the imposed shift in ATP—and, via Equation (4), in NADH—the phase organization of metabolic oscillations is redistributed, with different pools exhibiting distinct sensitivities to changes in the energetic–Ca^2+^ phase relationship.

Finally, we examined the effect of altering not only the phase but also the amplitude of the Ca^2+^ signal. Progressive reduction of Ca^2+^ oscillation amplitude leads to increased accumulation of metabolites in pool $P_{2}$, while pools $P_{1}$ and $P_{3}$ become increasingly depleted. As Ca^2+^ oscillations weaken, the amplitude of oscillatory carbon redistribution diminishes and the system approaches a regime characterized by elevated, near-steady-state levels in $P_{2}$ and reduced, weakly varying levels in $P_{1}$ and $P_{3}$. These results indicate that Ca^2+^ dynamics with sufficiently large oscillatory amplitude are a necessary condition for sustaining coordinated cataplerotic–anaplerotic cycling and phase-structured carbon redistribution within the DAM framework.
